# Welding Seam Trajectory Recognition for Automated Skip Welding Guidance of a Spatially Intermittent Welding Seam Based on Laser Vision Sensor

**DOI:** 10.3390/s20133657

**Published:** 2020-06-29

**Authors:** Gaoyang Li, Yuxiang Hong, Jiapeng Gao, Bo Hong, Xiangwen Li

**Affiliations:** 1 Key Laboratory in Hunan Provincial for Welding Robot and Its Application, Xiangtan University, Xiangtan 411105, China; gaoyang.li@cimc.com (G.L.); gaojiapeng_xtu@163.com (J.G.); xwlee@xtu.edu.cn (X.L.); 2 College of Mechanical and Electrical Engineering, China Jiliang University, Hangzhou 310018, China; hongyuxiang@cjlu.edu.cn

**Keywords:** automated skip welding, spatially intermittent welding seam, complex box girder structures, laser scanning displacement sensor, Euclidean distance discrimination

## Abstract

To solve the problems of low teaching programming efficiency and poor flexibility in robot welding of complex box girder structures, a method of seam trajectory recognition based on laser scanning displacement sensing was proposed for automated guidance of a welding torch in the skip welding of a spatially intermittent welding seam. Firstly, a laser scanning displacement sensing system for measuring angles adaptively is developed to detect corner features of complex structures. Secondly, a weld trajectory recognition algorithm based on Euclidean distance discrimination is proposed. The algorithm extracts the shape features by constructing the characteristic triangle of the weld trajectory, and then processes the set of shape features by discrete Fourier analysis to solve the feature vector used to describe the shape. Finally, based on the Euclidean distance between the feature vector of the test sample and the class matching library, the class to which the sample belongs is identified to distinguish the weld trajectory. The experimental results show that the classification accuracy rate of four typical spatial discontinuous welds in complex box girder structure is 100%. The overall processing time for weld trajectory detection and classification does not exceed 65 ms. Based on this method, the field test was completed in the folding special container production line. The results show that the system proposed in this paper can accurately identify discontinuous welds during high-speed metal active gas arc welding (MAG) welding with a welding speed of 1.2 m/min, and guide the welding torch to automatically complete the skip welding, which greatly improves the welding manufacturing efficiency and quality stability in the processing of complex box girder components. This method does not require a time-consuming pre-welding teaching programming and visual inspection system calibration, and provides a new technical approach for highly efficient and flexible welding manufacturing of discontinuous welding seams of complex structures, which is expected to be applied to the welding manufacturing of core components in heavy and large industries such as port cranes, large logistics transportation equipment, and rail transit.

## 1. Introduction

**Figure 1:**
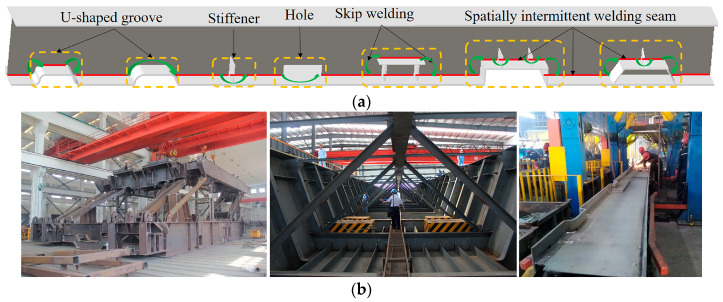
(**a**) Schematic diagram of skip welding; (**b**) typical example of spatial discontinuous welds in complex box girder.

Complex box girder components are critical components for equipment used in industries such as heavy machinery, special containers, ships, bridges, and heavy-duty vehicles [1,2,3,4]. Welding is the key technology for the manufacture of complex box girders [1,2,3,4]. Due to the complicated structures of complex box girders, e.g., U-shaped grooves, holes, and reinforcing plates used to reduce welding deformation, a welding seam trajectory is usually an intermittent spatial curve (Figure 1). Spatially intermittent welding seams usually need to be completed by varying positions and skip welding.

With the development of information sensing technology and modern manufacturing technology, automated, robotized, flexible, and intelligent welding manufacturing has become an inevitable trend [4,5,6]. Nevertheless, the current welding and manufacturing of complex box girders is still mainly completed manually and by mechanized welding equipment. Although welding robots have been applied to the welding of some box girder components [7,8], due to the complicated manufacturing process of complex box girders, the accuracy of assembly positioning can be guaranteed only with difficulty, especially when it comes to tailor welding and combination welding of large and heavy components. Therefore, without sufficient teaching time, the robotic welding of such components cannot be realized. With the disadvantages of low programming efficiency and lack of flexibility [9,10,11], it is difficult to adapt robotic welding to the efficient and flexible production of complex box girders in small quantities.

To overcome the difficulties encountered by welding robots in teaching and offline programming mode operations, many studies have been performed to explore methods that can make robotic welding simpler and more effective [12,13,14,15,16,17,18,19,20,21]. Among these methods, welding seam trajectory recognition is used for welding path planning and welding seam tracing is a significant method for improving the efficiency and flexibility of robotic welding.

Having been extensively applied to robotic welding due to its high accuracy and non-contact, vision sensing is considered one of the most promising welding seam trajectory recognition technologies [19,20,21,22,23,24,25,26,27,28,29,30,31,32,33,34,35,36,37,38,39,40,41,42,43,44]. Great attention has been paid to welding seam detection methods based on structured light vision sensing [20,21,22,23,24,25,26,27,28,29,30,31,32,33,34,35,36,37]. Hascoet et al. [23] first used a single-line laser vision sensor to detect V-shaped groove shape information, then generated a torch path based on this information, and finally proposed a welding strategy for automated welding of ships. Prasarn et al. [24] developed a robotic welding seam tracking system based on a crossed structured light vision sensor, achieving the tracking of butt-jointed seams on the V-shaped grooves of thick plates. Li et al. [25] developed a robotic welding tracking system based on a single-line laser vision sensor for the tracking of butt-jointed seams on the V-shaped groove of medium-thick plates of molten electrode gas shielded welding. Zeng et al. [26] integrated fusion light and structured light information to identify the positions of multi-layer/multi-pass welding (MLMPW) seams. Zou et al. [27] developed a robotic welding seam tracking system based on three-line laser vision to track the trajectory of the welding seams of complex curved overlap welding. Later, they detected the positions and characteristics of welding seams using a three-line laser vision sensor, and they proposed a real-time position estimation method [28] that improved the adaptability of a robotic welding position and the quality of complex curved welding. Zeng et al. [29] proposed a 3D path demonstration method based on an X-type laser vision sensor, achieving the 3D welding seam trajectory recognition of narrow butt-jointed seams. Zhang et al. [30] mounted a Keyence laser displacement sensor onto an industrial robot to effectively obtain the 3D information of a workpiece through multi-segment scanning. They used gray-scale image processing to extract the characteristics of the welding seams and they applied cubic smooth splines to reconstruct spatially complex curved welding seam models, accomplishing the detection of the characteristics of spatially complex curved overlap welding seams. Liu et al. [31] realized the three-dimensional reconstruction of the molten pool surface by projecting a 19-by-19 dot matrix structured light pattern on the weld pool area. Some studies explored welding seam trajectory recognition methods based on passive vision sensing technology [38,39,40,41]. By visually measuring the offset between the center of a welding seam and a welding gun, Ma et al. [38] proposed a welding seam tracking method that achieved the welding tracking of straight butt-jointed seams. Mitchell et al. [39] applied reliable image matching and triangulation algorithms to achieve the robust identification and positioning of Z-type and S-type narrow butt-jointed seams. Wang et al. [40] designed a measurement system that consisted of a multi-optical magnifier, a camera, and an external lighting diode lamp, and they devised a welding seam detection method based on narrow depth of field (NDOF) to accurately detect narrow welding seams in laser welding. In some studies, binocular vision sensing technology was used for the 3D reconstruction of welding seam trajectory. Mitchell et al. [42] installed two color cameras on the welding gun of an industrial robot, and they used the adaptive linear growth algorithm to identify the robustness of fillet welding seams. Drago et al. [43] applied a 3D vision detection system for the online detection of the 3D welding paths of arcs to automatically identify arc welding abnormalities. Yang et al. [44] designed a 3D structured light sensor using a DLP projector and two cameras to obtain 3D information of welding seams. However, the above studies were primarily focused on the trajectory recognition of continuous welding seams, and the sensors were required to be accurately and complexly calibrated before welding. Additionally, their image processing methods were complicated and time-consuming, showing poor robustness to the environment of the field. There is currently no study on the spatially intermittent welding seam trajectory recognition of complex welded structures.

A welding seam trajectory recognition method for automated skip welding guidance of spatially intermittent welding seams was proposed in this study. First, a laser scanning displacement sensing system with an adaptive measuring angle was designed in order to detect the features of the corners of complex structures in real time. Second, a welding seam trajectory recognition algorithm based on Euclidean distance discrimination was recommended in order to identify spatially intermittent welding seams. The algorithm extracted the shape features by constructing the characteristic triangles of the welding seam trajectory, and then discrete Fourier analysis was used to process the shape feature set to solve the feature vector that was used to describe the shape. Third, based on the Euclidean distance of the feature vector of the test sample and the class matching library, the class to which the sample belonged was discriminated, thereby achieving the online automated recognition of spatially intermittent welding seams. In this study, the laser scanning displacement sensing system was used to collect the trajectory samples of four typical spatially intermittent welding seams from 200 groups of complex box girder structures to test the classification accuracy of the classification model. To verify the effectiveness of the method, the test was conducted on the production line of foldable special containers.

The rest of the paper is structured as follows. Section 2 provides an overview of the experimental device used in this study, the principle of welding seam detection based on the experimental device, and the approach of recognizing the spatially intermittent welding seams. Section 3 describes the testing of the performance of the proposed method and the field testing of the automated skip welding of the foldable special container beam, and the test results are discussed. The conclusions are presented in Section 4.

## 2. Experimental Methods

### 2.1. Experimental Details

**Figure 2:**
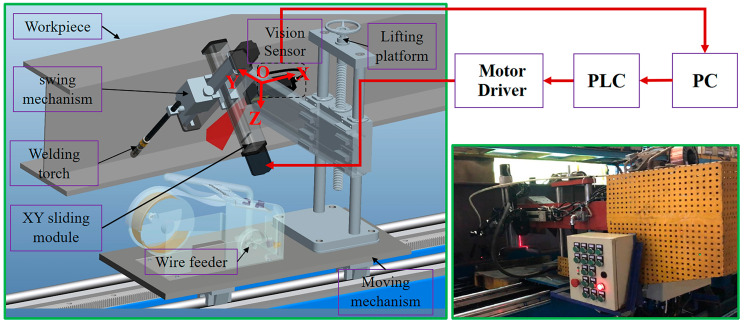
The configuration of the experimental system.

Figure 2 is an image of the experimental system and devices. The laser scanning displacement sensing system consisted of a laser generator (laser wavelength: 650 nm, power: 100 mw), a narrow band-pass filter (650 nm light waves could be passed), a CCD (TCD1208AP), a stepping motor, and receiving lens. Instead of being connected to the welding gun, the laser scanning displacement sensing system was installed a certain distance ahead of the welding gun, and the system was mainly used to detect the distance between the measurement point at the surface of the welding seam groove and the origin of the coordinates. The programmable logic controller (PLC) system was mainly used to control all of the operations that were required for the welding gun swing mechanism, XY sliding module, and moving mechanism to complete the welding process. The welding gun was controlled by the XY sliding module to move forward and backward. To control the welding gun in a cone-shaped swing, the welding gun swing mechanism was installed on the XY sliding module. The system device was driven by the moving mechanism to move along the welding direction. The lifting platform was used to adjust the overall large workpiece tooling drop before welding. For the PC, a CPU processor with a main frequency of 1.8 G (Intel (R) i5-8250, RAM 8 G) was used to run the main program algorithm and undertake complex computing tasks.

**Figure 3:**
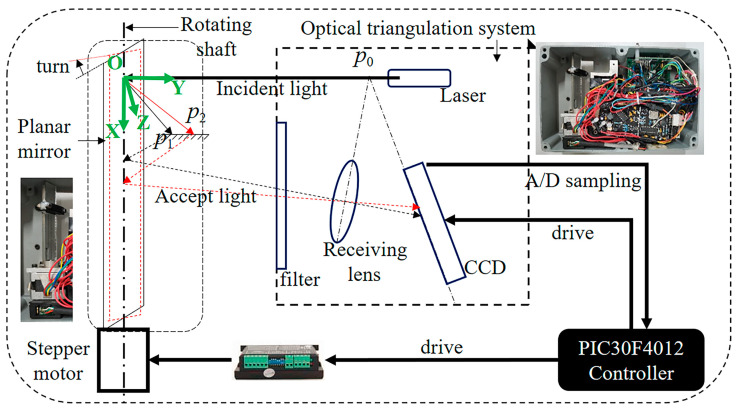
The configuration of the designed laser scanning displacement sensing system.

In this study, a laser scanning displacement sensing system was designed independently based on an improved laser displacement sensor at conventional points. Based on the optical triangulation system of the point laser displacement sensor, a rotary mirror device—including a motor and a plane mirror, as well as a motor that could drive the plane mirror to rotate coaxially—was added to the laser scanning displacement sensing system. The rotary mirror device could change the measurement direction of the optical triangulation system, whereas the conventional point laser optical triangulation system could only measure the distance in the direction of the laser beam. Hence, the designed sensing system could measure in multiple directions with the physical position and attitude of the system being constant, greatly improving the measurement degree of freedom of the sensor (Figure 3). As shown in Figure 3, this sensing system was mainly composed of a point laser optical triangulation system and a rotary mirror device. The motor and the plane mirror were coaxial, with the angle between the axis of the emitted laser and the rotation axis of the stepping motor being 90°. During the measurement, the stepping motor was controlled to drive the deflection of the plane mirror, which deflected the incident laser beam, thereby changing the measurement direction of the optical triangulation system. The measurement principle was as follows: first, the laser beam that was emitted by the optical triangulation system was reflected by the plane mirror onto the workpiece. Then, the laser spot on the surface of the workpiece was reflected by the plane mirror to the optical triangulation system. Ultimately, the length of the incident laser beam (e.g., $Op_{2}$ and $Op_{1}$) at different deflection angles could be solved for based on the principle of triangulation.

### 2.2. Corner Position and Trajectory Detection of Fillet Welds

**Figure 4:**
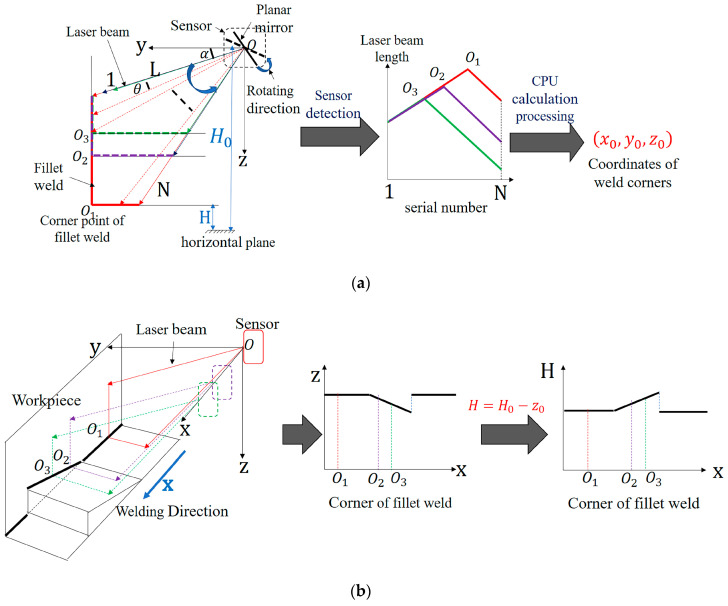
Schematic diagram of seam position and trajectory detection: (**a**) weld position detection; (**b**) weld trajectory detection.

**Figure 5:**
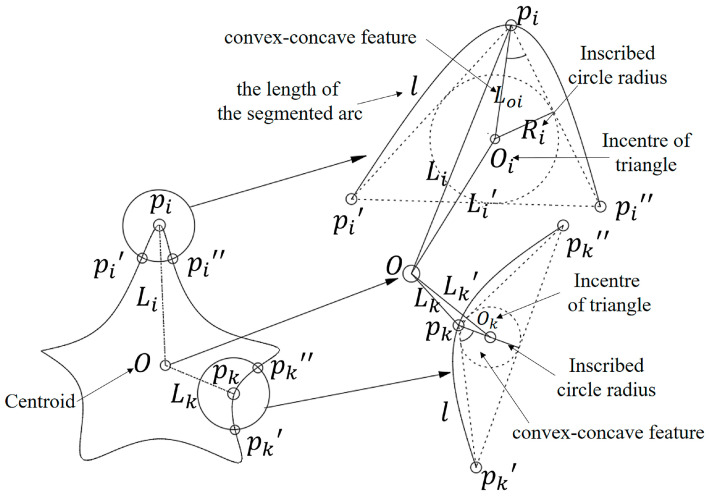
Definition of characteristic parameters of a seam trajectory.

Figure 4 shows a schematic diagram of welding position and trajectory detection. A coordinate system was established with the intersection point of the plane mirror axis of the laser scanning displacement sensing system (i.e., the axis of the motor shaft) and the incident laser axis as the origin O. The direction of the plane mirror axis (i.e., the welding direction) was established as the X axis, and the perpendicular lines pointing from the origin to the surface of the right-angle welding seam groove were established as the Y and Z axes. As shown in Figure 4a, the rotary mirror device of the laser scanning displacement sensing system drove the measuring to rotate counterclockwise at the starting angle α, measure once for each rotation at a certain angle of θ, and then transmit the distance information of the measurement point to the PC processor in real time. The laser scanning displacement sensing system returned to its original position after rotating and measuring N times, and no measurement was performed during this reset process. During the reset process of the sensor, the PC processor used the difference method to process the above-mentioned measured data to obtain the distance information of the measurement points near the welding seam corner. In the end, $\left(x_{0},y_{0},z_{0}\right)$, the coordinates of the corner of the fillet welding seam, were calculated based on the distance information and corresponding deflection angle. As shown in Figure 4b, when the device moved along the welding direction, the corner trajectory of a right-angle welding seam was detected, and the two-dimensional trajectory of this corner in the XZ plane was the object extracted and analyzed in this study. To facilitate the analysis, the variation trend of the horizontal height H (the reference height from the welding seam corner to the level ground) was used to characterize the two-dimensional welding seam corner trajectory in the XZ plane. As shown in Figure 5, the equation for solving the horizontal height H was

(1) {x0=S y0=Lcos(nθ+α) n=1,2,3,⋯,Nz0=Lsin(nθ+α) n=1,2,3,⋯,NH=H0−z0 

where s denotes the moving length of the system device, L is the distance from the origin O to the measurement point on the surface of the welding seam groove (i.e., the length of the incident laser beam), $H_{0}$ denotes the reference height from the origin O to the level ground, n is the serial number of the measurement point, N refers to the number of measurements within a scanning cycle, θ is the angle between the adjacent measurement laser beams, and α is the angle between the initial measurement laser beam and the Y axis.

### 2.3. Seam Trajectory Features Extraction

The contour curve of an object has two shape features, i.e., the inscribed radius and the concave-convex feature of a characteristic triangle. The characteristic triangle was constructed as follows: the contour curve was sampled uniformly. Two points, i.e., $p_{i}{}^{\prime }$ and $p_{i}{}^{\prime \prime }$, were found clockwise and counterclockwise along the contour curve, with the random point $p_{i}$ being the starting point. l (known as the length of the segmented arc) denoted the length of the arcs between the two points and $p_{i}$, and a characteristic triangle $\Delta p_{i}{}^{\prime }p_{i}p_{i}{}^{\prime \prime }$ was formed by the description of point $p_{i}$ for the shape of the contour curve. As shown in Figure 5, $p_{i}$ and $p_{k}$ were two random sampling points on the contour curve, O was the shape center of the contour, $\Delta p_{i}{}^{\prime }p_{i}p_{i}{}^{\prime \prime }$ and $\Delta p_{k}{}^{\prime }p_{k}p_{k}{}^{\prime \prime }$ were the characteristic triangles at the sampling points $p_{i}$ and $p_{k}$, $O_{i}$ and $O_{k}$ represented the incentre of $\Delta p_{i}{}^{\prime }p_{i}p_{i}{}^{\prime \prime }$ and $\Delta p_{k}{}^{\prime }p_{k}p_{k}{}^{\prime \prime }$. $L_{i}$ and $L_{k}$ referred to the distances from O to $p_{i}$ and $p_{k}$, $L_{i}{}^{\prime }$ and $L_{k}{}^{\prime }$ were the distances from O to $O_{i}$ and $O_{k}$, $L_{Oi}$ and $L_{Ok}$ were the distances from $p_{i}$ and $p_{k}$ to $O_{i}$ and $O_{k}$, and $R_{i}$ and $R_{k}$ were the inscribed circle radiuses of $\Delta p_{i}{}^{\prime }p_{i}p_{i}{}^{\prime \prime }$ and $\Delta p_{k}{}^{\prime }p_{k}p_{k}{}^{\prime \prime }$.

**Figure 6:**
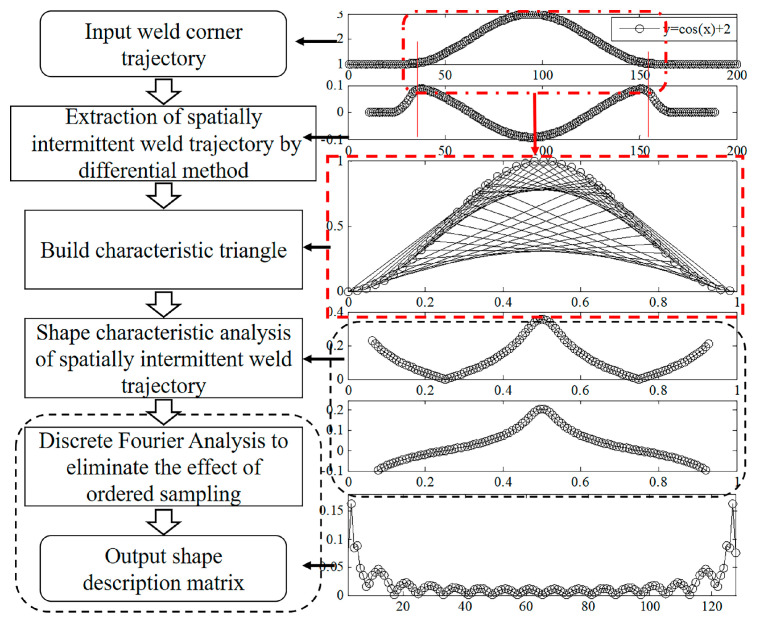
The processing result of each step of the proposed features extraction algorithm.

The shape analysis of trajectory of the spatially intermittent welding seam corner consisted of three parts. First, the difference method was used to analyze the welding seam corner trajectory to extract data about the trajectory of the spatially intermittent welding seam corner. Second, the shape features of the trajectory of the spatially intermittent welding seam corner were extracted. Third, a Fourier analysis was conducted on the set of shape features, and then the Fourier shape descriptor of the trajectory of the spatially intermittent welding seam corner was solved. Figure 6 shows the flow chart of the shape analysis of the cosine curve. The difference method was first applied to extract the trajectory of the spatially intermittent welding seam corner. The characteristic triangle was then established based on the shape of the trajectory of the spatially intermittent welding seam corner, and two shape features (i.e., the inscribed radius and degree of concavity-convexity) were extracted. To eliminate the impact of the starting point, a set of shape features was formed based on the plural form of the two shape features. The set was then processed by discrete Fourier analysis to solve for the feature vector (also known as the Fourier shape descriptor) that was used to describe the shape. Finally, the category of the samples was determined based on the Euclidean distance between the feature vector of the test sample and the class matching library.

$P=\left\{P_{1},\;P_{2},\;\;P_{3},\;\cdots ,\;\;P_{i},\cdots ,\;P_{N}\right\}$ was the welding seam trajectory, so the difference value $\Delta_{i}$ between the left and right differences at $P_{i}$ could be expressed by Equation (2).

(2) $$\Delta_{i}=\frac{\left(P_{i+m}+\cdots +P_{i+1}-mP_{i}\right)-\left(mP_{i}-P_{i-m}-\cdots -P_{i-1}\right)}{m},\;m\leq i\leq N-m$$

In the equation, $P_{i+m}+\cdots +P_{i+1}-mP_{i}$ is the backward difference of $P_{i}$, $mP_{i}-P_{i-m}-\cdots -P_{i-1}$ is the forward difference of $P_{i}$, and m is the length of the data traversed by the difference method. A reasonable m could eliminate noise interference.

A differential analysis was performed on the trajectory of spatially intermittent welding seam corner based on Equation (2), the first extreme point $P_{a}$, and the last extreme point $P_{b}$ after the differential analysis was solved. Then the welding seam trajectory interval [a,b] was where the shape feature needed to be extracted.

The inscribed radius was the ratio of the area to the half-perimeter of the characteristic triangle. The inscribed radius was mainly used to describe the global features of the shape at point $p_{i}$. As shown in Figure 5, based on Heron’s formula, Equation (3) for the inscribed radius $R_{i}$ of the characteristic triangle at point $p_{i}$ was derived:

(3) $$R_{i}=\frac{\sqrt{c_{i}\left(c_{i}-l_{i}{}^{\prime }\right)\left(c_{i}-l_{i}{}^{\prime \prime }\right)\left(c_{i}-l_{i}{}^{\prime \prime \prime }\right)}}{c_{i}}$$

where $R_{i}$ is the inscribed radius of the characteristic triangle $\Delta p_{i}{}^{\prime }p_{i}p_{i}{}^{\prime \prime }$ at point $p_{i}$, $l_{i}{}^{\prime }$, $l_{i}{}^{\prime \prime }$ and $l_{i}{}^{\prime \prime \prime }$ refer to the distance from point $p_{i}$ to point $p_{i}{}^{\prime }$, the distance from point $p_{i}$ to point $p_{i}{}^{\prime \prime }$ and the distance from point $p_{i}{}^{\prime }$ to point $p_{i}{}^{\prime \prime }$, respectively, and $c_{i}$ denotes the half-perimeter of the triangle.

The concave-convex feature was a product of the concavity and convexity of the shape multiplied by the concave-convex height. As shown in Figure 5, the concavity and the convexity of the shape was defined as follows: when the distance $L_{i}$ between the contour centroid O and point $p_{i}$ was greater than the distance between the contour centroid O and the inner center $O_{i}$ of the characteristic triangle $\Delta p_{i}{}^{\prime }p_{i}p_{i}{}^{\prime \prime }$, the contour shape at point $p_{i}$ was convex with respect to the centroid, with the concavity and convexity of the shape being positive. In contrast, when the distance $L_{i}$ between the contour centroid O and point $p_{i}$ was smaller than the distance between the contour centroid O and the inner center $O_{i}$ of the characteristic triangle $\Delta p_{i}{}^{\prime }p_{i}p_{i}{}^{\prime \prime }$, the contour shape at point $p_{i}$ was concave when compared with the centroid, with the concavity and convexity of the shape being negative.

The concave-convex height was defined as follows: $L_{Oi}$ was the distance between the sampling point $p_{i}$ and the inner center $O_{i}$ in the characteristic triangle $\Delta p_{i}{}^{\prime }p_{i}p_{i}{}^{\prime \prime }$. The greater the $L_{Oi}$ was, the steeper the slope of the shape was. The smaller the $L_{Oi}$ was, the gentler the slope of the shape was.

Therefore, the equation for solving the degree of concave-convex feature (CCH) was

(4) $$CCH_{i}=\{\begin{array}{c} L_{Oi}\;\;\;\;\;,\;\;\;L_{i}-L_{i}{}^{\prime }>0\\ 0\;\;\;\;\;\;\;\;,\;\;\;L_{i}-L_{i}{}^{\prime }=0\\ -L_{Oi}\;,\;\;\;L_{i}-L_{i}{}^{\prime }<0 \end{array},\;\;\;i=1,2,3,\cdots ,N$$

To orderly sample the contour curve, an appropriate starting point needed to be selected. When the starting point of the sampling point set changed, the sampling point set experienced a translation, and only the phase of the corresponding Fourier transform coefficient changed accordingly, with its amplitude value remaining constant. Hence, with only the amplitude of the Fourier coefficient being used to describe the final shape feature, the influence of the position of the starting point on the Fourier shape descriptor was eliminated. Therefore, the inscribed radius $R_{i}$ that was normalized at all sampling points was combined with the concave-convex feature $CCH_{i}$ in the form of a complex number $z_{i}=R_{i}+CCH_{i}j$ to finally obtain a set of complex numbers (hereinafter referred to as the shape feature complex function) corresponding to the set of contour sampling points that could characterize the shape features. The real part of the complex function represented the sampling points, description of the global shape feature of the contour curve, and the imaginary part characterized the detailed shape features of the contour curve at the sampling points.

A discrete Fourier transform was performed on the shape feature complex function, and the Fourier transform coefficient was calculated using the following equation:

(5) $$Z_{k}=\frac{1}{N}\overset{N}{\underset{i=1}{\sum }}\left(R_{i}+CCH_{i}j\right)exp\left(\frac{-j2\pi ki}{N}\right),\;\;\;i=1,2,3,\cdots ,N$$

where $Z_{k}$ denotes the Fourier transform coefficient of the shape feature complex function, N represents the number of sampling points, and k is a frequency domain variable.

The modulus of the Fourier transform coefficient $Z_{k}$ that was obtained with Equation (5), $\left|Z_{k}\right|$, was calculated, and the Fourier shape descriptor W was finally solved, the equation of which was

(6) $$W=\left[\;\left|Z_{1}\right|\;\left|Z_{2}\right|\;\left|Z_{3}\right|\cdots \left|Z_{k}\right|\;\right]$$

where k is a frequency domain variable, $\left|Z_{k}\right|$ is the modulus of the Fourier transform coefficient with k as the frequency domain variable, and W is the Fourier shape descriptor.

### 2.4. Classification Method Based on Euclidean Distance

The category of the test samples in the category matching library was determined by the Euclidean distance. The similarity between the test samples and the category matching library was measured with the Euclidean distance. Distance and similarity were inversely related. Apparently, the category most similar to the category matching library was the category to which the test samples belonged. The calculation equation was as follows:

(7) $$d\left(W_{X},\;W_{Ci}\right)=\sqrt{\overset{m}{\underset{k=1}{\sum }}{\left(Z_{Xk}-Z_{Cik}\right)}^{2}}$$

(8) $$C_{X}=argmin\left\{d\left(W_{X},\;W_{Ci}\right),\;\;\;i=1,2,3,\cdots ,N\right\}$$

where $W_{X}$ is the Fourier shape descriptor of test sample X, $W_{Ci}$ is the Fourier shape descriptor of category $C_{i}$ in the category matching library, m is the frequency domain variable of the Fourier analysis, $Z_{Xk}$ and $Z_{Cik}$ are the moduli of the k-th Fourier transform coefficients of $W_{X}$ and $W_{Ci}$, and $C_{X}$ denotes the category to which the test samples belong.

The category matching library was obtained by training the samples using the least square method. First, the above method was used to solve the Fourier shape descriptor of the training samples. The optimal solution matching the Fourier shape descriptor of the training samples was calculated using the least square method, and this optimal solution was then used as the matching library of this category. The relevant derivation equation was as follows:

(9) $$E=\overset{N}{\underset{i=1}{\sum }}{\left(W_{y}-W_{i}\right)}^{2}$$

(10) $$W_{y=C}=argmin\left\{E\right\}$$

where E denotes the squared loss function of the least square method, $W_{y=C}$ is the optimal solution of the training samples that was calculated using the least square method, $W_{i}$ is the Fourier descriptor of the i-th training sample, $W_{y}$ is the Fourier descriptor of the matching test, and N is the number of training samples.

## 3. Results and Discussion

### 3.1. Results of 3D Trajectory Detection and Corner Trajectory Shape Feature Extraction of Welds

**Figure 7:**
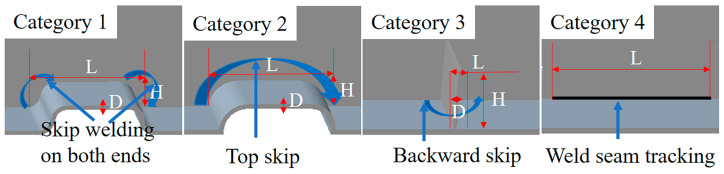
Schematic diagram of welding treatment for Categories 1, 2, 3, and 4.

**Figure 8:**
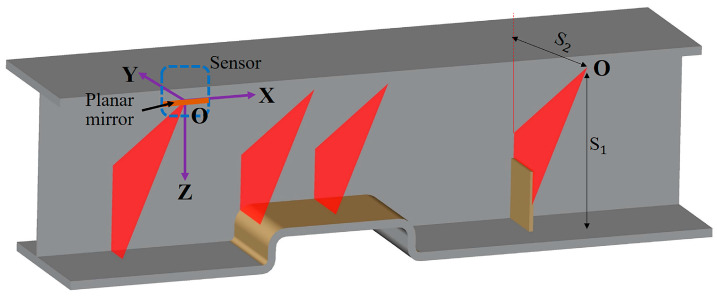
Laser displacement sensor detection workpieceα.

Figure 7 shows the four types of trajectory shapes used in this study, which were Categories 1, 2, 3, and 4, the most commonly used categories in the welding structure of complex box girders. The criteria for discriminating between these categories were determined by the shape of the welding seam trajectory and the welding process. As shown in Figure 8, Category 1 was S-shaped at both ends and straight at the top. When Category 1 was welded, the welding gun skipped the S-shaped ends to weld on the straight part at the top. Category 2 was arc-shaped at both ends and straight at the top. For the welding of Category 2, the welding gun skipped the top to weld on the normal welding section at the back. Category 3 was generally used to prevent the welding from deforming into a rectangular shape. When Category 3 was welded, the welding gun skipped Category 3 to weld on the normal welding section at the back. Category 4 was normal welding. For the welding of Category 4, the welding seam tracking was required during the welding process. Considering the welding speed, the measurement range of the laser scanning displacement sensor, the advanced scanning distance of the laser scanning displacement sensor, the size parameters of the four categories needed to be within specific applicable ranges (Table 1).

As shown in Figure 4 and Figure 8, a coordinate system was established with the intersection point between the rotary mirror axis (i.e., the axis of the motor shaft) and the incident laser axis as the origin O. The plane mirror axis (i.e., the welding direction) was established as the X axis, and the perpendicular lines pointing from the origin to the surface of the right-angle welding seam groove were established as the Y and Z axes. At the starting position detected by the system device, the vertical distances from the origin O to the surface of the fillet welding seams in the Y and Z directions were 400 and 410 mm, respectively, and the horizontal height $H_{0}$ between the origin O and the level ground was 580 mm. The laser scanning displacement sensor scanned counterclockwise, with the angle α between the initial measurement laser beam and the Y axis being 33.024°, the angle θ between adjacent measurement laser beams being 0.5°, and the number N of measurements being 40.

**Figure 9:**
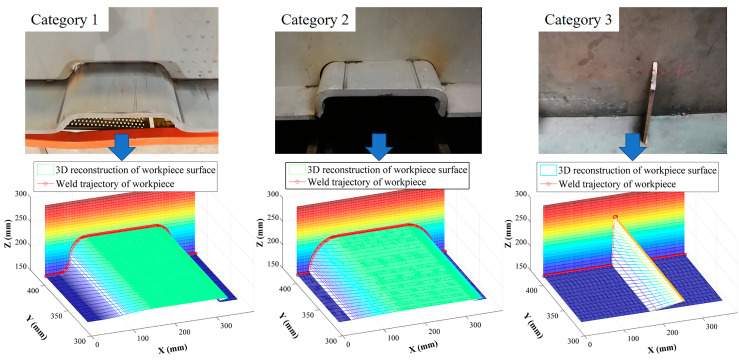
3D reconstruction results of welding seam.

**Figure 10:**
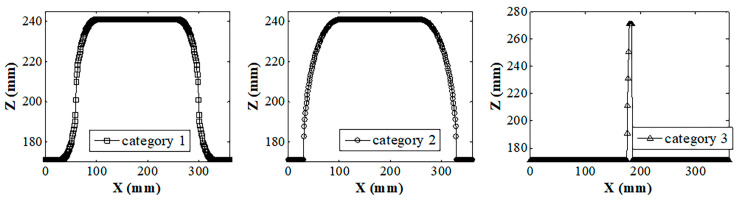
Weld corner trajectories of Categories 1, 2, and 3.

Figure 9 shows the 3D reconstruction results for the system’s detection of the welding seam trajectory in the moving state, for which the moving speed (i.e., the welding speed) was 1.2 m/min, the moving length was 350 mm, and the scanning frequency of the laser scanning displacement sensor was 20 Hz. Category 1 had a size of 300×70×16(L×H×D), Category 2 had a size of 300×70×16(L×H×D), and Category 3 had a size of 5×120×5(L×H×D). Figure 10 shows the welding seam corner trajectories of Categories 1, 2, and 3 that were extracted using the difference method.

**Figure 11:**
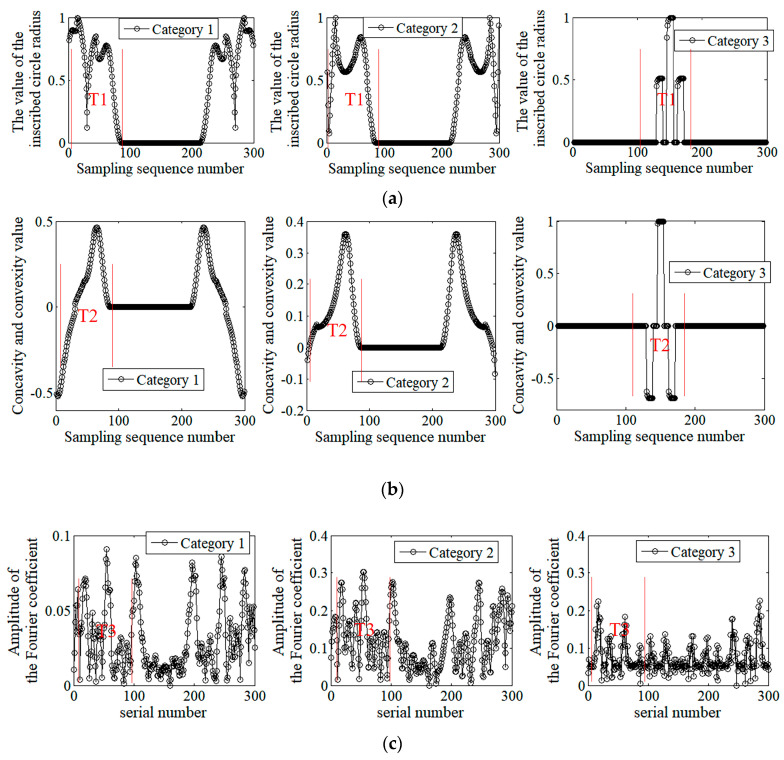
Shape extraction results for corner trajectories of welds in Categories 1, 2, and 3: (**a**) inscribed circle radius; (**b**) concavity and convexity; (**c**) Fourier shape descriptors.

Figure 11 shows the shape features of the welding seam corner trajectory shown in Figure 10 that were extracted using the method mentioned in Section 2, with a segmented arc length of 20 sampling intervals (the sampling interval in this article is 1 mm). Figure 11a shows the shape feature curve represented by an inscribed radius. Figure 11b shows the shape feature curve characterized by the degree of concavity–convexity. Figure 11c shows the Fourier shape descriptor that was solved using the shape feature extraction method. By comparing the T1, T2, and T3 areas in Figure 11a–c, it was found that the extracted shape features could effectively reflect the differences between the welding seam corner trajectories of Categories 1, 2, and 3.

**Figure 12:**
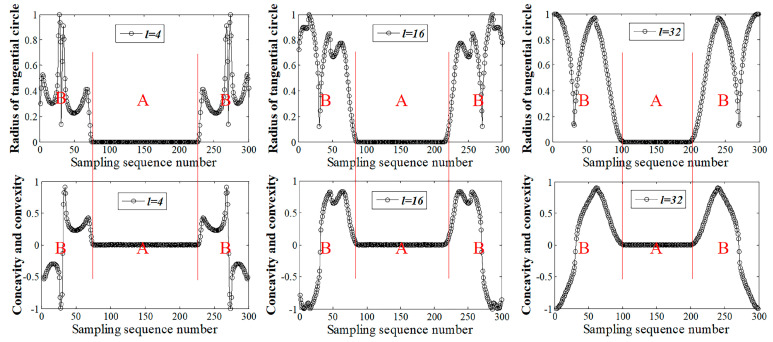
Shape characteristic curves under different segmentation arc lengths.

With the welding seam corner trajectory of Category 1 in Figure 11a as the case, the corresponding characteristic triangles for the shape feature extraction were constructed with the length of the segmented arc being four sampling intervals, 16 sampling intervals, and 32 sampling intervals, the results of which are shown in Figure 12. In Figure 12, the upper side shown is the shape feature curve represented by the inscribed radius of the characteristic triangle, the lower side shown is the shape feature curve represented by the degree of concavity-convexity, area A shows the top straight part of Category 1, and area B shows the S-shaped curve part at both sides of Category 1.

As shown in Figure 12, when the length of the segmented arc of the characteristic triangle was four sampling intervals, area B of the shape feature curve experienced a large fluctuation, while area A was a straight line with a value of 0. When the length of the segmented arc was 16 sampling intervals, area B of the shape feature curve had small fluctuations, and area A was less wide than when the length of the segmented arc was four sampling intervals. When the length of the segmented arc was 32 sampling intervals, area B of the shape feature curve fluctuated rarely, and area A was shorter than when the length of the segmented arc was four or 16 sampling intervals. All these phenomena implied that when the segmented arc was relatively short, the shape feature curve mainly revealed local detailed features, while when the segmented arc was comparatively long, the curve reflected more about the global shape features. Hence, the segmented arc of the characteristic triangle needed to be neither too long nor too short, so that both the local details and the global shape features could be fully reflected. By comprehensively considering the sampling interval and the trajectory scale, the length of the segmented arc l of the characteristic triangle was expressed as

(11) $$l=\frac{2^{T}}{2^{3}}\varepsilon +\delta ,\;\;\;\;2^{5}\leq 2^{T}\leq NUM,\;0<\delta \leq 10$$

where l is the length of the segmented arc of the characteristic triangle, NUM denotes the number of sampling points (NUM is close to the value of $2^{T}$), δ is the empirical compensation value, and ε represents the sampling interval.

### 3.2. Classification Experiment Based on the Euclidean Distance

To verify the effectiveness of the proposed method in recognizing the welding seam trajectories of Categories 1, 2, and 3, some experiments were carried out. The data for the welding seam trajectories that were detected in the experiments served as the test sample for classification. The experimental device performed detection based on the established coordinate system shown in Figure 8. The experimental parameters are shown in Table 2. As shown in Table 2, S_1_ and S_2_ refer to the vertical distances from the origin O to the surface of the welding seam grooves in the Y and Z axes, α is the angle between the initial measurement laser beam and the Y axis, and *θ* is the angle between adjacent measurement laser beams. N denotes the number of measurements of the sensor within one cycle, V is the moving speed of the system device, and F is the scanning frequency of the laser scanning displacement sensor. The laser scanning displacement sensor scanned counterclockwise, with the horizontal height *H*_0_ from the origin O to the level ground being 580 mm. Category 1 had a size of 300×70×16(mm), Category 2 had a size of 300×70×16(mm), and Category 3 had a size of 5×120×5(mm), with 22 sampling intervals being the length of segmented arc that constituted the characteristic triangle.

**Figure 13:**
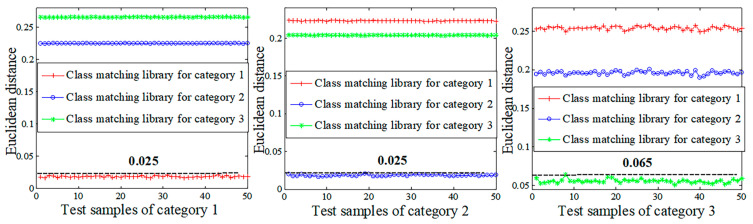
Classification test results for Categories 1, 2, and 3 test samples.

**Figure 14:**
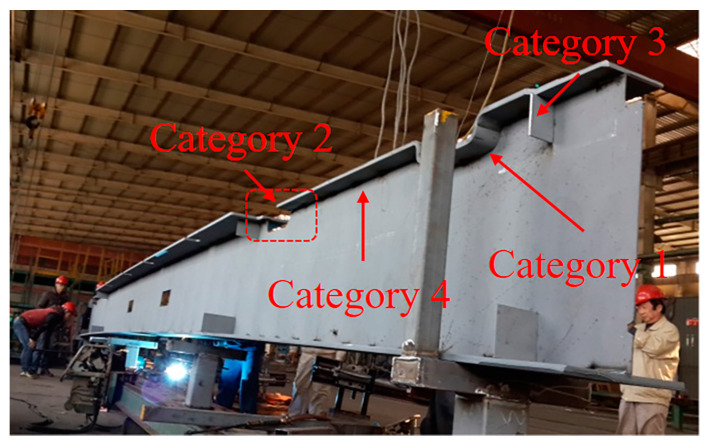
Example of workpiece of the folding special container girder.

Ten sets of welding seam trajectory data were randomly selected from the data of the welding seam trajectories of Categories 1, 2, and 3. These data sets were used as training samples for solving the classification and matching library using the least square method. Fifty sets of data about the welding seam trajectories were selected as the test samples to calculate the Euclidean distance between the test samples and the matching library they belonged to. The results of this calculation are shown in Figure 13. As shown in Figure 14, the Euclidean distance between both of the test samples in Categories 1 and 2 and the matching library to which they belonged was less than 0.025, and the Euclidean distance between the test samples in Category 3 and the matching library to which they belonged was less than 0.065. All of the test samples in the 150 sets had the smallest Euclidean distance with the matching libraries that they belonged to, indicating that the proposed method could accurately recognize 100% of the test samples in the 150 sets.

The processing time of this algorithm was measured by functions in MATLAB. The measurement results were as follows: (1) the communication time between the sensor and the PC did not exceed 15 ms. (2) The real-time extraction of the welding seam corner trajectory did not exceed 30 ms. (3) The analysis and classification concerning the shapes of the trajectories did not exceed 20 ms. Therefore, the total time cost did not exceed 65 ms, satisfying the needs of online identification for the types of welding seam trajectory during the welding process. The experimental results showed that this method could be used to accurately detect the positions of the 3D welding seams and to identify the welding seam corner trajectory in the real time during the process of welding the structure made of complex box girders.

### 3.3. Welding Experimental and Field Test Validation

**Figure 15:**
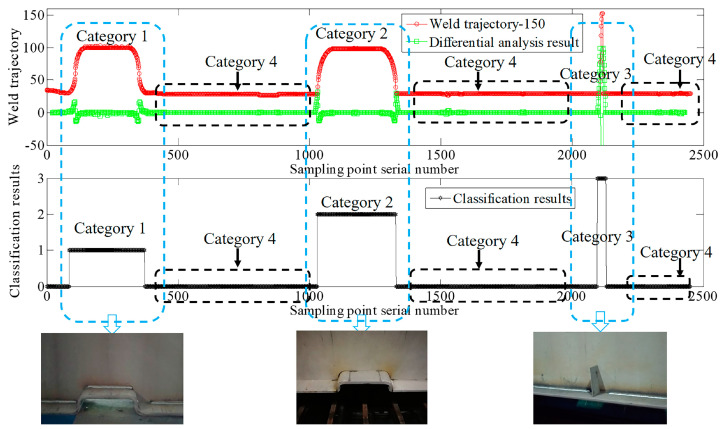
Results of weld trajectory identification and automated skip welding in field tests.

The most common workpiece of the foldable special container was selected as the test sample to be used in the field test. The workpiece was 14,000 mm long, 300 mm wide, and 500 mm high (Figure 14). A coordinate system was established for the experimental device, as shown in Figure 8. On the YZ plane, S_2_ and S_1_, the vertical distances from the origin O to the surface of the welding seam grooves in the Y and Z directions, were 390 and 400 mm, respectively. The horizontal height $H_{0}$ from the origin O to the level ground was 590 mm and the distance from the origin O to the welding gun was 500 mm. In terms of the welding direction, the laser scanning displacement sensor moved along the direction driven by the moving mechanism at a speed of 1.2 m/min (i.e., the welding speed), with the moving length being 2500 mm. The laser scanning displacement sensor scanned counterclockwise, with the scanning frequency being 20 Hz, the angle α between the initial measurement laser beamand the Y axis being 32°, the angle θ between the adjacent measurement laser beams being 0.5°, and the number N of measurements being 40. The welding voltage was 30 V, the welding current was 320 A, and the welding protective gases were carbon dioxide and argon mixed at a ratio of 1:4. Figure 15 shows the experimental results that were obtained based on the above parameters.

As shown in Figure 15, the laser scanning displacement sensor detected the distance between the origin O and the measuring point on the surface of the workpiece’s fillet welding seam groove and the sensor sent the measured distance to the PC processor in real time. Once the PC processor received the distance data for one scanning cycle, the difference method was used to solve the coordinates of the fillet welding seam. In this way, the system device could acquire data about the fillet welding seam corner trajectory in real time during mobile welding, and the system device could perform real-time differential analysis on this welding seam corner trajectory to extract data about the welding seam corner trajectories in Categories 1, 2, and 3. Then the shape features of this welding seam corner trajectory were analyzed and classified using the method proposed in this study. According to the results of online classification, the PC processor first created sub-welding tasks in the trajectory segment where the class is located (sub-welding tasks of Categories 1, 2, and 3 are shown in Figure 7), and then inserted the sub-tasks into the corresponding guide trajectory of the welding torch, finally controlled the PLC controller to drive the four-axis robot to guide the welding torch to perform the jump welding operation. As shown in Figure 15 and Table 3, the proposed method welded the workpiece at a high speed of 1.2 m/min using the correct welding processing based on the identification of the fillet welding seam corner trajectory, verifying the effectiveness of the proposed method in practical application. As required by the on-site manufacturing process, a certain margin without welding was left at both ends of the test samples in Category 2.

Based on the field welding test results, the laser scanning displacement sensing system that was developed in this study was able to scan the workpiece in real time during the welding process and to guide the welding gun in order to complete the automated skip welding of spatially intermittent welding seams. Without any requirement for calibration and additional offline or online teaching programming, the system device that was developed based on this sensing system greatly improved the efficiency of the welding manufacturing. When the spatial trajectories of large and complex structures are scanned, the welding seam trajectory is likely to shift beyond the measurement range of a laser displacement sensor, which places high demands on the measurement range and degree of freedom of a sensor. The laser scanning displacement sensing system that was developed in this study not only inherited the advantages of the point laser displacement sensing method, but also could adaptively change the viewing angle without changing the physical position or posture by changing the deflection angle of the rotary mirror, remarkably improving the measurement degree of freedom of the sensing system. Moreover, in this sensing system, the laser spot on the axis of the laser beam can be clearly imaged on the line array CCD. Through the conventional filtering method and centroid method, the influence of surface reflection can be eliminated, and the imaging position of the laser spot can be extracted. This is one of the factors of strong anti-interference ability of this measurement method. Compared to the sensing method in this study, the single-line laser vision sensing method limits the measurement range and measurement freedom due to factors such as the depth of field and angle of view of the camera lens, and the laser streak and the reflection streak are imaged together in the camera when detecting highly reflective workpieces (e.g., aluminum plate), and a complex algorithm is needed to filter out the artifacts caused by reflection in the image. Of course, the single-line laser vision sensing method also has great advantages, such as high detection efficiency, small structure, and high two-dimensional measurement resolution. Therefore, if there are large measurement ranges, reflection phenomena, low measurement frequency requirements, and low cost requirements in the application scenario, it is recommended to select the point laser + rotating mirror method for detection. If there are narrow butt joints, narrow groove welds, or the need to obtain groove shape information in the detection scene, it is recommended to use single-line laser visual sensing to detect.

The advanced scanning distance of the laser scanning displacement sensor proposed in this study could be theoretically set to 350 mm. Since the on-site manufacturing process required a certain margin without welding to be left at both ends of the test samples in Category 2, the advanced scanning distance was actually set to 500 mm. This advanced scanning distance was mainly determined by two factors, i.e., the structural size of the spatially intermittent welding seams and the time consumed by the welding seam trajectory recognition algorithm. If the distance was too long, the structure of the system device would be enlarged, and the real-time storage pressure of the PC database would be increased. If the distance was too short, the welding seam trajectory might be incorrectly identified. In actual application, this distance can be changed according to the site conditions.

This paper proposes a seam trajectory recognition algorithm based on Euclidean distance discrimination. In this paper, the algorithm takes less than 65 ms. The time consumed by the algorithm increases with the number of classes in the class matching library. Therefore, in the future, we will try to further improve the classification library model based on efficient intelligent machine learning algorithm to adapt to more types of complex welding structure products in heavy machinery and other fields.

## 4. Conclusions

In order to realize the automated skip welding guidance of complex box girder structures, a method for detecting the corner trajectories and identifying the spatially intermittent welding seams was proposed, and a hardware system supporting this method was established.

A laser scanning displacement sensor with adaptive field of view was proposed. The sensor is based on the optical design of a combination of a point laser type optical triangulation and a rotating mirror, which can change the measurement angle while maintaining the physical installation position and attitude of the sensor, so as to adapt to the workpiece weld corners detection under different product types and complex conditions.

A weld trajectory recognition algorithm based on Euclidean distance discrimination was proposed. The algorithm extracts the shape features by constructing the characteristic triangle of the weld trajectory, and then processes the set of shape features by discrete Fourier analysis to solve the feature vector used to describe the shape. Finally, based on the Euclidean distance between the feature vector of the test sample and the class matching library, the class to which the sample belongs is identified. The classification accuracy rate of the algorithm for four kinds of the spatially intermittent welding seams in common complex box girder structures is 100%. The overall processing time for weld trajectory detection and classification does not exceed 65 ms.

The field test was carried out on the special type container girder production line. The results show that the system proposed in this paper can accurately identify the discontinuous welds in the high-speed MAG welding process with a welding speed of 1.2 m/min, and guide the welding torch to automatically complete “welding-arc extinguishing-obstacle avoidance-arc starting-welding”, which significantly improved the welding manufacturing efficiency and quality stability of complex box girder components. This method does not require time-consuming pre-weld teaching programming and visual inspection system calibration. It provides a new technical approach for the efficient and flexible welding of discontinuous welds with complex structures, and provides a key technical basis for a new generation of intelligent robot welding systems.

In order to further specifically optimize and expand the applicability of the method, future work needs to improve the intelligent classification library based on efficient machine learning algorithms to achieve stable, fast, and accurate online discrimination of more types of weld trajectories, and is expected to be applied to the welding and manufacturing of core components of major industries such as port cranes, large logistics transportation equipment, and rail transit.

## Figures and Tables

**Table 1:** Table of dimensional parameters for Categories 1, 2, 3, and 4.

Classification	Measurement (mm)	Welding Treatment
Category 1	L×H×D:(100×50×16, 400×100×16)	Top welding
Category 2	L×H×D:(100×50×16, 400×100×16)	Top skip
Category 3	L×H×D:(5×50×5, 15×120×15)	Backward skip
Category 4	L:(30, 450)	Weld seam tracking

**Table 2:** Experimental parameters of Categories 1, 2, and 3 weld trajectory detection.

Experiment Number	S_1_ (mm)	S_2_ (mm)	α	θ	N	F (Hz)	V (m/min)	Measurement Quantity
Experiment 1	380–430	400	33°	0.5°	40	16	1.0	Category 1: 20
								Category 2: 20
								Category 3: 20
Experiment 2	400	380–430				16	1.0	Category 1: 20
								Category 2: 20
								Category 3: 20
Experiment 3	400	400				15–20	1.0	Category 1: 10
								Category 2: 10
								Category 3: 10
Experiment 4	400	400				20	0.8–1.3	Category 1: 10
								Category 2: 10
								Category 3: 10
Experiment 5	400	400	33°	0.2°–0.8°	40	20	1.2	Category 1: 10
								Category 2: 10
								Category 3: 10

**Table 3:** The key parameters and test results of the field test.

The Advanced Detection Distance of the Sensor (mm)	Welding Speed (mm/s)	Scanning Frequency (Hz)	Classification Errors
500	20	20	0
